# Specific Primers and Nested PCR Find *Trichophyton rubrum* Missed by Culture of Ground Toenails from Onychomycosis in Podiatric Patients in Eastern Australia

**DOI:** 10.3390/jof11070520

**Published:** 2025-07-14

**Authors:** Anjana C. Santosh, Danilla Grando, Ann C. Lawrie

**Affiliations:** School of Science, RMIT University, Bundoora, Melbourne 3083, Australia; anjanasanthosh147@gmail.com (A.C.S.); danilla.grando@rmit.edu.au (D.G.)

**Keywords:** Onychomycosis, tinea unguium, tinea pedis, dermatophytes, *Trichophyton interdigitale*, *Trichophyton rubrum*, podiatry, PCR

## Abstract

Toenail onychomycosis causes significant problems in public health and is more common among the elderly and immune-compromised populations. A previous culture-based survey of communal finely ground toenails from the east coast of Australia isolated 125 *T. interdigitale* but only one *T. rubrum*. This paucity of *T. rubrum* was surprising because it is one of the most common dermatophytes isolated worldwide. Our aim was to find out if *T. rubrum* was present but not cultured. DNA was extracted from ground toenails from the same samples. New specific primers were designed for the ITS region of *T. rubrum* that excluded *T. interdigitale* and vice versa. PCR with these new primers found *T. rubrum* as well as *T. interdigitale* in all ground toenail samples. This suggests that *T. rubrum* was present and common in the ground toenails. It was possibly missed by culture because it grows slowly and was overgrown by *T. interdigitale* and non-dermatophyte moulds. Alternatively, its viability may have declined earlier, during collection, treatment, or storage of the ground toenails. This has implications for studies of clinical materials, especially nails, as infection by *T. rubrum* (the most common dermatophyte) may be missed by culture, the main method used in pathology laboratories.

## 1. Introduction

### 1.1. Onychomycosis, Symptoms, and Cost

Onychomycosis and tinea pedis of the foot are common diseases worldwide and are economically important [1,2]. Worldwide, 85–90% of tinea occurs in the foot, in the skin (tinea pedis), and in the toenails (tinea unguium, onychomycosis) [2,3]. Both tinea unguium and onychomycosis can lead to serious thickening and deformity of the nails, with significant cosmetic, clinical, and economic consequences. The incidence increases with age (50% of cases are over 60 years old), gender (males more than females), and health (one-third of cases are diabetic) [2,3]. In patients with impaired peripheral circulation and nerve damage, such as diabetics, painless subungual ulcers can develop, requiring surgical intervention and hospitalisation [2,4,5].

Onychomycosis and tinea pedis are economically important. Worldwide, they account for nearly half of all nail-related consultations [6] and about 90% of dermatophyte infections [5]. The incidence in the population has been estimated as 7–25% [2]. In 2021, the direct cost of dermatophytosis (ringworm) in the USA alone was estimated at USD 845 m [1]. In Australia, hospitalisation of diabetics, mainly for foot-related problems and their complications, was estimated to cost about AUD 48 m/year in 2010 [4]. These costs would be expected to increase along with the spread and severity of symptoms due to more aggressive and more frequently antifungal-resistant dermatophytes or non-dermatophyte moulds (NDMs), such as has occurred with *T. indotineae* in India [1,7].

### 1.2. Nail Sampling and Causative Fungi

Nails suspected of disease are sampled either by clipping and scraping darkened and distorted parts of the nail or by high-speed electric burring [2,8]. Burring, most commonly practised by podiatrists, collects material into a bag containing 100–200 mL of finely ground toenails from up to 500 patients. Viable fungi from the nails can be isolated and cultured on selective media using standard methods [2,9,10].

Onychomycosis of the toenails (especially the great toenail) can involve three types of fungi: dermatophytes (which are presumptively pathogenic), yeasts, and non-dermatophyte moulds (NDMs), often accompanied by bacteria [2,11,12]. The most common infecting agents are two anthropophilic dermatophytes in the genus *Trichophyton*: *T. rubrum* and *T. interdigitale* (the latter in the *T. mentagrophytes* complex) [13], which are found in 50–90% of symptomatic toenails in all habitable continents except parts of Africa [5,8,14]. Less common dermatophytes associated with onychomycosis include *Epidermophyton floccosum* (now generally less than 1% of aetiological agents) and the rare *Paraphyton cookei* [10,11,14,15].

Storage, especially cold storage, can affect the viability of fungi in the clinical material [16]. The anthropophilic *T. rubrum* and *T. interdigitale* were viable for only 7 and 9 months, respectively, in human skin scales stored dry at 18–25 °C [17]. By contrast, the zoophilic *T. mentagrophytes* showed no decrease in viability when infected pig scales were stored dry in the dark for 6 months at 4–30 °C [18]. Storage also resulted in increases in the isolation of non-dermatophyte moulds (NDMs), yeasts, and bacteria [19].

### 1.3. Molecular Approaches

The poor recovery rate of dermatophytes by culture from infected clinical material is well documented [3,5,8]. DNA-based assays have generally improved dermatophyte detection by up to double that of culture [8,20,21,22,23,24], and various authors have called for the routine use of DNA-based assays to improve detection and diagnosis time [2,25]. Dermatophytes have also been found using DNA-based methods in culture-negative nails, in the presence of other fungi, and in dual dermatophyte infections, to the extent that ITS-based PCR has been proposed as the gold standard for dermatophyte diagnosis [8,26]. Some authors have called for a combination of histopathology and PCR to replace wet-prep and culture as the gold standard [8,21,27]. Using a combination of culture and PCR can distinguish between viable and inviable *T. rubrum*; for example, *T. rubrum* declined to only 19% from clinical skin and nail samples stored at 4–8 °C for 10–32 months [16], but PCR with *T. rubrum*-specific primers detected *T. rubrum* in 100% of the samples before storage and 92% after storage.

DNA extracted from the ground toenails can be searched for DNA of *T. rubrum* by using specific primers or probes to discriminate between *T. rubrum* and *T. interdigitale* [22,25,28,29]. The amplicons, of up to 700 bases, can be searched for closest matches through GenBank, available through NCBI (National Center for Biotechnology Information) (https://www.ncbi.nlm.nih.gov/). The sensitivity of PCR can be increased further by ‘nesting’ specific primers to detect *T. rubrum* and/or *T. interdigitale* in clinical specimens [19,20,30,31].

### 1.4. Problem and Aims

Hainsworth et al. [10] recently isolated 125 *T. interdigitale*, one *P. cookei,* and surprisingly, only one *T. rubrum* from podiatric samples of ground toenails stored at 4 °C for up to 6 months. Samples were spread over DTM (dermatophyte test medium) [32] and dermatophytes isolated by picking out white/cream colonies with a characteristic red reverse on DTM. *Paraphyton cookei* is rare [14,33], and its paucity was expected. However, *T. rubrum* is one of the two most common dermatophytes in nails worldwide, including Australia [2], and its paucity was unexpected. Hainsworth et al. [10] attributed this imbalance between *T. rubrum* and *T. interdigitale* to the historical predominance of *T. interdigitale* isolates from toenails from older patients in Melbourne, Australia [34], who constitute a large proportion of podiatry patients. Nevertheless, the very low frequency of *T. rubrum* in this analysis was concerning and suggested that the collection method or storage of the ground nails before isolation and culture could have affected the viability of *T. rubrum* more than that of *T. interdigitale*. *Trichophyton rubrum* could be present but not cultured due to damage during grinding [35], cold storage [16], or overgrowth by other fungi [5]. Molecular approaches have the potential to detect if *T. rubrum* is present but not viable in the specimens studied previously by Hainsworth et al. [10].

The aim of this study was to determine if *T. rubrum* was present in the ground toenails by extracting DNA from the same ground toenails as sampled by Hainsworth et al. [10] in a culture-based study. In this study, we report that ITS-based specific primers found both *T. rubrum* and *T. interdigitale* in all samples, despite *T. interdigitale* being almost exclusively the sole dermatophyte found by culture.

## 2. Materials and Methods

Seventeen cultures of seven authenticated dermatophyte species were obtained from the RMIT Fungal Culture Collection (RFCC) and ATCC (American Type Culture Collection) (through In Vitro Technologies Pty Ltd., Noble Park North, VIC, Australia) (Table S1). Samples of finely ground toenails were provided by Steven Hainsworth from nine bags: three from each of the Bass Coast (R4, R5, R33), Metropolitan Melbourne (R8, R9, R35), and Rural Victoria–Shepparton (R34, R38, R40). Samples from these same bags had already resulted in the isolation of 125 cultures of *T. interdigitale* but only one *T. rubrum* (from Bag R38) [10]. DNA was extracted from 100 mg of each fungus or ground toenails using a Qiagen DNeasy Plant Mini Kit (Qiagen, Clayton, Victoria, Australia), according to the manufacturer’s instructions.

For PCR, the universal primers ITS1 or ITS5 (forward) were first used with the ITS4 (reverse) primer [36] to verify that the DNA extracts could amplify in PCR. Each 25 μL PCR reaction contained 12.5 μL of GoTaqGreen Master Mix (Promega, Alexandria, NSW, Australia), 8.5 μL of nuclease-free water (NFW, Peoria, IL, USA), 1 μL of 10 μM ITS1 or ITS5 primer, 1 μL of 10 μM ITS4 primer, and 2–5 μL of DNA extract. Thermocycling was 94 °C for 10 min; 35 cycles of: 94 °C for 30 s, 51 °C for 30 s, 72 °C for 1 min; and a final 72 °C for 10 min.

Amplicons produced were sequenced to check that each of the named species of fungi had the expected closest match. PCR products were purified with a Qiagen QIAquick PCR Purification Kit and sequenced using the BigDye Terminator v3.1 Cycle Sequencing Kit protocol (Applied Biosystems, Foster City, CA, USA), followed by electrophoresis at Micromon, Monash University, Clayton, Victoria (https://platforms.monash.edu/micromon/). The ITS sequences of the closest BLAST matches and corresponding Type species were downloaded from the National Center for Biotechnology Information (NCBI) (https://www.ncbi.nlm.nih.gov/), and phylogenetic trees were constructed in MEGA Version 7 (https://www.megasoftware.net/dload_win_gui).

To design specific primers for *T. rubrum* and *T. interdigitale*, the ITS sequences of the type species of *T. rubrum* and *T. interdigitale* (downloaded from GenBank through NCBI) were aligned in MEGA7 with sequences of authenticated dermatophytes used in this study and with the closest matches downloaded from GenBank. Primers that theoretically should react with only one species were designed manually and tested for specificity by BLAST searching on NCBI. These new specific primers were a reverse primer TrRev (5′-CCTGAGGGCGCTGAATTGGC-3′) for *T. rubrum* and a reverse primer TmiRev (5′-CCTGGAGGCGCTGGTTTGTTG-3′) for *T. interdigitale*. The primers were synthesised by IDT (Integrated DNA Technologies, Clayton, Victoria, Australia).

For PCR with the specific reverse primers, each was paired with the universal forward primer ITS5. Each PCR reaction contained 12.5 μL of GoTaqGreen Master Mix (Progen), 7.0 μL of sterile Milli-Q water, 2.5 μL of 10 μM ITS5 (forward) universal primer, 1 μL of 25 μM TmiRev or TrRev (reverse) primer, and 2–5 μL of DNA extract. Thermocycling, electrophoresis, and imaging were as before. ‘Nested’ PCR was used to confirm reactions and for sequencing. First-round PCRs were performed using the universal primers ITS5 + ITS4; second-round PCRs used ITS5 with either TrRev or TmiRev, except that 2 μL from the first ITS5 + ITS4 PCR was used instead of 2 μL of DNA extracts.

## 3. Results

### 3.1. Testing of Specific Primers with Extracts of Pure Cultures

**Figure 1:**
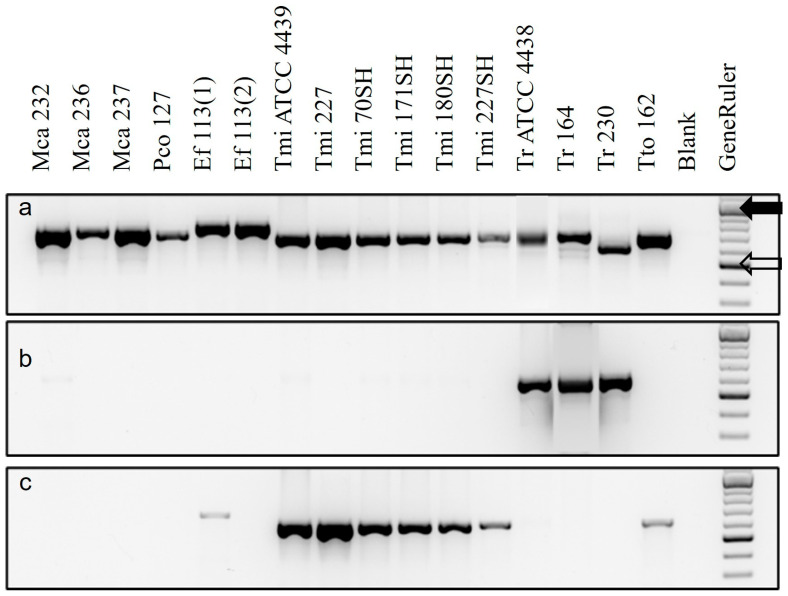
PCR results from pure fungal cultures using primer ITS5 (forward) paired with reverse primer (**a**) ITS4, (**b**) TrRev or (**c**) TmiRev. Key: Mca = *Microsporum canis*, Pco = *Paraphyton cookei*, Ef = *Epidermophyton floccosum*, Tmi = *Trichophyton interdigitale*, Tr = *T. rubrum*, Tto = *T. tonsurans*, Blank = no DNA, GeneRuler (solid arrow = 1000 bp, hollow arrow = 500 bp). Numbers refer to strains in RFCC or ATCC.

PCR with ITS5 + ITS4 universal primers of all DNA extracts from pure cultures resulted in ITS sequences with the closest matches (≥99%) to their expected identities (Figure 1a, Table S2). All sequences were uploaded into GenBank (Accession numbers OP271463–271471). The reverse primer TrRev reacted with *T. rubrum* but not with *T. interdigitale* (Figure 1b), while the reverse primer TmiRev reacted with *T. interdigitale* and *T. tonsurans* but not with *T. rubrum* (Figure 1c). Both TrRev and TmiRev produced a faint band with *E. floccosum* but not with *M. canis* or *P. cookei*. Thus, the specific primers reacted as expected to discriminate between *T. rubrum* and *T. interdigitale*.

### 3.2. Use of Specific Primers and Nested PCR with Ground Toenail Extracts

**Figure 2:**
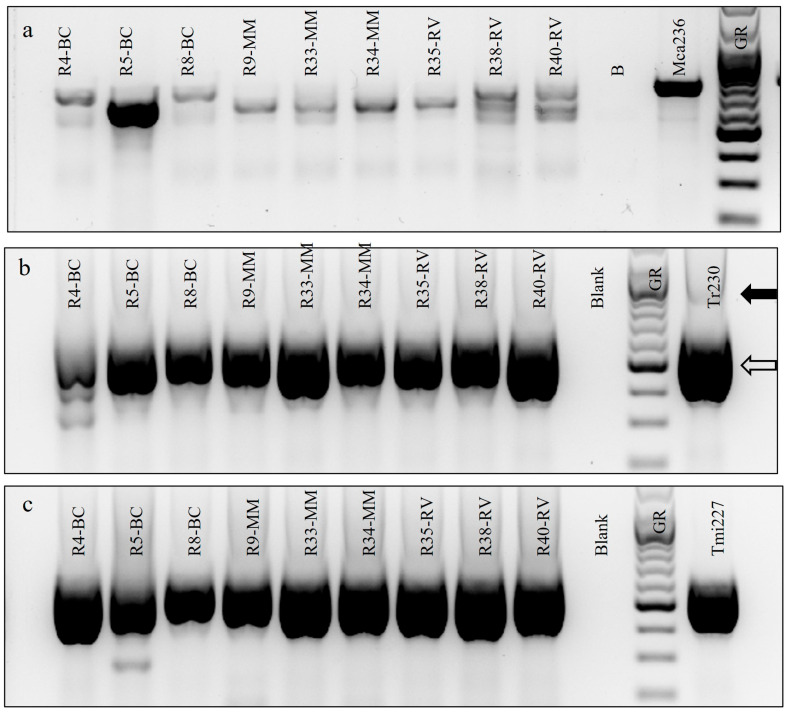
PCR results from ground toenail bags with (**a**) initial primers ITS5 + ITS4, followed by nested primer ITS5 (forward) paired with reverse primer (**b**) TrRev or (**c**) TmiRev. Key (left to right): R4–R40 = ground toenail sample bags; BC = Bass Coast, MM = Metropolitan Melbourne, RV = Rural Victoria; Blank = no DNA, GeneRuler (solid arrow = 1000 bp, hollow arrow = 500 bp), Mca = *Microsporum canis* (in (**a**)), Tr230 = *Trichophyton rubrum* (in (**b**)), Tmi227 = *T. interdigitale* (in (**c**)). Numbers refer to strains in RFCC. B = Blank (no DNA) in (**a**).

With universal primers, DNA extracts from only three ground toenail samples produced strong single bands (Figure 2a). The ITS sequences from Bags R4 and R38 matched >99% to *T. rubrum* in GenBank, and that from Bag R5 matched 99% to that of the xerophile *Aspergillus penicillioides* (Table S2). Subsequent culture of ground toenails from Bag R5 on the low a_w_ medium ATCC MY50G [37] resulted in the growth of abundantly sporing *A. penicillioides*.

**Figure 3:**
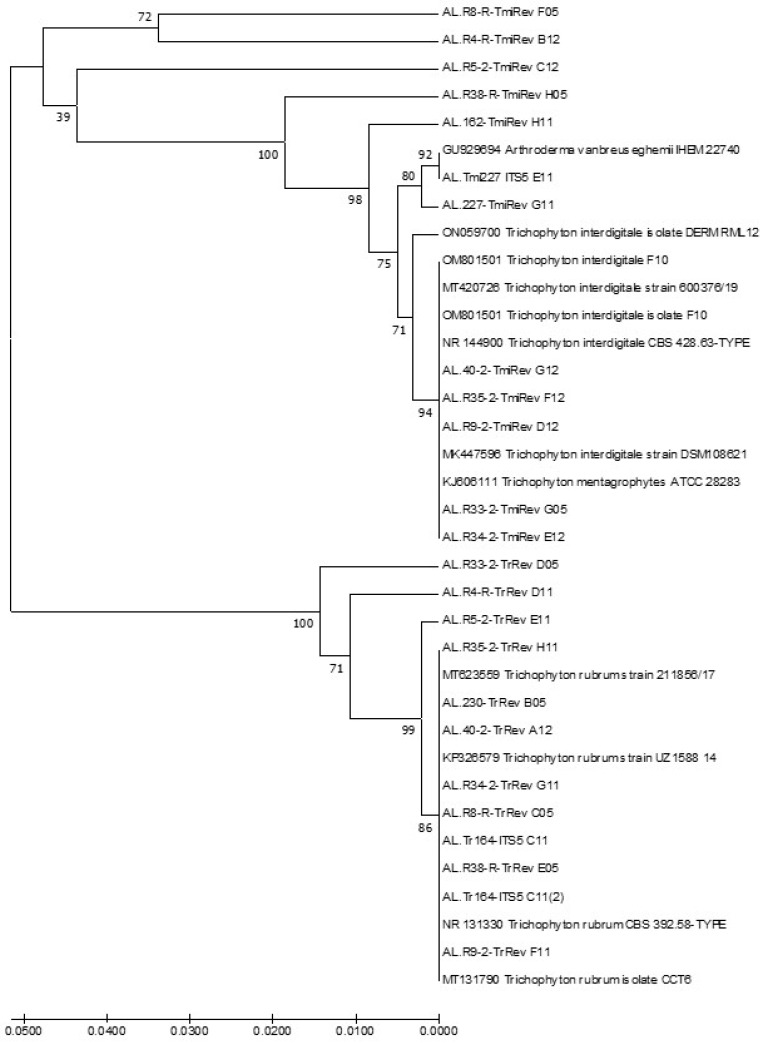
Relationships between ITS sequences of ground toenails and those of the type species and closest matches for two *Trichophyton* species. This evolutionary history was inferred using the UPGMA method [38]. The optimal tree with the sum of branch length = 0.29289323 is shown. The percentages of replicate trees in which the associated taxa clustered together in the bootstrap test (500 replicates) are shown next to the branches [39]. The tree is drawn to scale, with branch lengths in the same units as those of the evolutionary distances used to infer the phylogenetic tree. The evolutionary distances were computed using the Jukes–Cantor method [40] and are in the units of the number of base substitutions per site. The analysis involved 36 nucleotide sequences. All positions containing gaps and missing data were eliminated. There were 479 positions in the final dataset. Evolutionary analyses were conducted in MEGA7 [41].

Priming the DNA extracts from the remaining ground toenail samples with either ITS5 + TrRev or ITS5 + TmiRev resulted in fainter bands that were undesirable for direct sequence with universal primers (Figure 2a). However, when the PCR products from ITS5 + ITS4 (universal) primers were used as the first pair of primers and either ITS5 + TrRev (Figure 2b) or ITS5 + TmiRev (Figure 2c) as the second, both resulted in strong single bands that were sequenced. The sequences of products from ITS5 + TrRev matched sequences of *T. rubrum,* and those from ITS5 + TmiRev matched those of the *T. mentagrophytes/interdigitale* complex (Table S3). When these sequences were aligned with those from the type species and those of the closest matches, they clustered with those of *T. rubrum* or the *T. mentagrophytes/interdigitale* complex, respectively (Figure 3).

## 4. Discussion

This study provides evidence that both *T. interdigitale* and *T. rubrum* were present (live or dead) in all ground toenail samples. These new specific primers found *T. rubrum* DNA in the presence of *T. interdigitale* DNA and vice versa. This differs from previous results from the culture of the samples, which isolated *T. interdigitale* from all but one bag but isolated *T. rubrum* only once [10]. It does, however, fit with an average of 21% *T. rubrum* found in DNA extracted simultaneously from samples of the same ground toenails by metagenomics [42] and dominance by equal numbers of *T. rubrum* and *T. interdigitale* in entire nails [12].

This corrects the previous report that *T. rubrum* was absent or rare in the symptomatic ground toenails, although CFUs (culture-forming units) of *T. interdigitale* may have been more abundant, resulting in its greater isolation frequency; this could be investigated by qPCR. It is, therefore, important from a clinical and epidemiological viewpoint. It also supports suggestions that DNA-based methods should be used along with histology to check for non-viable or slower-growing dermatophytes [2,33,43].

The results from this study confirm those of three previous studies in south-eastern Australia, which demonstrated that *T. mentagrophytes* and *T. rubrum* were the principal pathogens in toenail infections in three cities in Australia: Melbourne [34], Sydney [44], and Adelaide [45]. Furthermore, equal quantities of DNA of both *T. interdigitale* and *T. rubrum* were found by metagenomics in samples of symptomatic nails collected by clipping and scraping [39]. This was as expected, as these two *Trichophyton* species are the two main pathogens involved in tinea unguium and onychomycosis worldwide [2,3,11,20].

### 4.1. Comparison of Methods

In general, little success by culture (10–30% detection) in the detection of dermatophytes has been found relative to DNA-based methods [2,3,15,19,22,46]. However, no one method here found all four dermatophytes in the bags (Table S4), suggesting that a mixture of microscopy, culture, and DNA-based methods is required for full exploration of potential pathogens [2]. Both culture [10] and specific primers (this study) found *T. interdigitale* in the ground toenails. However, culture much underestimated the expected incidence of *T. rubrum* (only one culture from Bag R38), whereas DNA-based PCR found *T. rubrum* in all bags. *Paraphyton cookei* was found only by culture, although its detection would be unlikely without suitable primers for PCR [45].

The faint cross-reactions of *E. floccosum* with both TrRev and TmiRev primers meant that its presence could not be excluded. However, *E. floccosum* was not found in culture (Hainsworth et al. 2020) [10] and has been isolated previously from only 0.43% of patients with tinea unguium in Melbourne and its surrounding areas (Coloe and Baird 2010) [34]. Also, Muir et al. [44] found no toenail infection caused by *E. floccosum* in a Sydney-based study. Worldwide, the incidence of *E. floccosum* in nails is relatively small and appears to be declining [5,11,34,44].

Both specific primers found and amplified their target species despite the relatively large amount of *Aspergillus penicillioides* in Bag R5. *Aspergillus penicillioides* is not a recorded cause of onychomycosis, although it comprised 9% of hits in symptomatic toenails by metagenomics [11]. It is best known for its growth only in low a_w_ environments (it is a xerophile) [37] and is most likely to have been present in the ground toenails and to have grown through them during storage at 4 °C before analysis [18,19]. It did not grow on the DTM used for isolation but did grow on the ATCC low a_w_ medium MY50G [37].

### 4.2. Problems with Trichophyton rubrum Detection

The underrepresentation of *T. rubrum* using isolation and culture relative to specific primers and metagenomics suggests that the *T. rubrum* DNA was from fungi that were mostly not viable when the samples were initially tested. Possible explanations centre around the initial collection of the samples and their storage: mechanical damage to the cell walls during collection [35], sensitivity to cold and desiccation during storage [16], and relatively slow growth compared with *T. interdigitale* and non-dermatophyte moulds [10] during isolation.

During collection, routine high-speed grinding during podiatry treatment of diseased nails may have damaged *T. rubrum* more than *T. interdigitale* because of differences in their cell wall ultrastructure. Chitin ‘rodlets’ (microfibrils) in the cell walls of *T. rubrum* are more exposed than in *T. interdigitale* [47], potentially resulting in greater loss of viability during collection. Corroborating this, viability in *T. rubrum* was reduced in nails by drilling rather than curettage during specimen collection [9,35], possibly due to a greater probability of cell wall damage. Heikkila [46] suggested that the heat generated during drilling could reduce viability in dermatophytes, as the temperature could reach 56 °C.

Subsequent storage of the ground toenails, especially at 4 °C, may also have contributed to greater loss of viability in dermatophytes, particularly *T. rubrum,* compared with other fungi [16,17,48]. Although cold storage may have further reduced the viability of all fungi, and disproportionately that of *T. rubrum* [16], it suggests that there was little viability in *T. rubrum* even when it was isolated, stored, and cultured soon after collection.

Overgrowth and destruction by other fungi during storage or isolation procedures may also have reduced viability in the dermatophytes [19]. It is also possible that *T. interdigitale* may have overgrown the tardier *T. rubrum* in cultures, as *T. interdigitale* grows at almost twice the rate of *T. rubrum*, and both fungi have been detected together in nail samples using specific primers [19,22,29,49].

It seems unlikely that the isolation medium (DTM) used previously by Hainsworth et al. [10] was biassed against *T. rubrum*, as Taplin et al. [32] stated that all dermatophytes tested grew well on it. Also, DTM has much in common with Sabouraud’s dextrose agar, which had a bias towards *T. rubrum* and away from *T. interdigitale* [19]. In addition, two previous studies that used DTM to isolate fungi found more *T. rubrum* than *T. interdigitale* [50,51], and DTM provided greater sensitivity and accuracy for dermatophyte primary isolation than other media [52].

### 4.3. Interpretation of Results

Caution is required in interpreting these results—finding DNA of a fungus in the nails is not the same as finding a live fungus [20,21]. Culture finds only fungi that are still alive, whereas DNA-based methods find amplifiable fragments of fungal DNA. The results from culture and DNA-based methods, such as specific primers, are thus complementary rather than contradictory and have, in this study, compared with Hainsworth et al. [10], distinguished between viable and non-viable dermatophytes in the samples.

Fungi vary in survival during storage, with survival of *T. rubrum* (and possibly *E. floccosum*) poorer than that of *T. mentagrophytes*, as found previously by others [16,17]. This aligns with *T. interdigitale* being found through culture and specific primers, while *T. rubrum* is only found by specific primers. The survival time of the geophilic *P. cookei* is unknown, but its culture from the ground toenails suggests that it was greater than that of the anthropophilic *T. rubrum*. It therefore seems likely that all four dermatophytes (*T. interdigitale, T. rubrum, P. cookie,* and possibly *E. floccosum*) were originally present (Table S4) but that only *T. interdigitale* commonly retained viability in the samples before and after storage. This could be investigated further by extracting RNA and searching for *T. rubrum* transcripts relative to those from *T. interdigitale*.

## 5. Conclusions

The DNA-based method of using specific primers found both *T. interdigitale* and *T. rubrum* in finely ground toenails from which only *T. interdigitale* was previously found by culture. This suggests that *T. rubrum* in most samples was not viable, due to damage during sample collection, sensitivity to cold during storage, or overgrowth by non-dermatophyte moulds. Further research is required to resolve these questions. The invaluable insights offered here by coupling results from a DNA-based method with those from culture suggest that thorough analysis of such clinical materials is needed to elucidate the pathogenesis of onychomycosis. More quantitative information may be obtained by using metagenomics in future.

## Data Availability

ITS sequences of all fungi used (Accession numbers OP271463–271471 made available on 27 August 2022) have been uploaded into GenBank via the portal at the National Center for Biotechnology Information (NCBI) (https://www.ncbi.nlm.nih.gov/).

## Supplementary Materials

The following supporting information can be downloaded at: https://www.mdpi.com/article/10.3390/jof11070520/s1, Table S1. Cultures used during this research. Table S2. Closest matches of ITS sequences in Blast searches on NCBI using universal primers ITS5+ITS4. Table S3. Closest matches of sequences in Blast searches on NCBI using new specific primers. Table S4. Relative discovery of dermatophytes in these ground toenails by different methods.

## Abbreviations

The following abbreviations are used in this manuscript:

ATCC	American Type Culture Collection
NCBI	National Center for Biotechnology Information
NDM	Non-dermatophyte mould
PCR	Polymerase chain reaction
RFCC	RMIT Fungal Culture Collection

## References

[1] CDC (Centers for Disease Control and Prevention) Impact of Fungal Diseases in the US. 2025. Data and Statistics on Fungal Diseases|Fungal Diseases|CDC. https://www.cdc.gov/fungal/data-research/facts-stats/index.html.

[2] Gupta A.K., Wang T., Cooper E.A., Lincoln S.A., Foreman H.-C., Scherer W.P., Bakotic W.L. (2024). Clinical diagnosis and laboratory testing of abnormal appearing toenails: A retrospective assessment of confirmatory testing for onychomycosis in the United States, 2022–2023. J. Fungi.

[3] Ghannoum M.A., Hajjeh R.A., Scher R.N., Konnikov N., Gupta A.K., Summerbell E., Sullivan S., Daniel R., Krusinski P., Fleckman P. (2000). A large-scale North American study of fungal isolates from nails: The frequency of onychomycosis, fungal distribution, and antifungal susceptibility patterns. J. Am. Acad. Dermatol..

[4] Thomas J., Jacobson G.A., Narkowicz C.K., Peterson G.M., Burnet H., Sharpe C. (2010). Toenail onychomycosis: An important global disease burden. J. Clin. Pharm. Ther..

[5] Gupta A.K., Stec N., Summerbell R.C., Shear N.H., Piguet V., Tosti A., Piraccini B.M. (2020). Onychomycosis: A review. J. Eur. Acad. Dermatol. Venereol..

[6] Fernández-Torres B., Cabañes F.J., Carrillo-Muñoz A.J., Esteban A., Inza I., Abarca L., Guarro J. (2002). Collaborative evaluation of optimal antifungal susceptibility testing conditions for dermatophytes. J. Clin. Microbiol..

[7] Bishnoi A., Vinay K., Dogra S. (2018). Emergence of recalcitrant dermatophytosis in India. Lancet Infect. Dis..

[8] Nenoff P., Klonowski E., Urlaß S., Verma S.B., Mayser P. (2023). Clinical picture, causative agents and diagnostics of dermatomycoses. Dermatologie.

[9] English M.P., Atkinson R. (1973). An improved method for the isolation of fungi in onychomycosis. Br. J. Dermatol..

[10] Hainsworth S., Hubka V., Lawrie A.C., Carter D., Vanniasinkam T., Grando D. (2020). Predominance of *Trichophyton interdigitale* revealed in podiatric nail dust collections in Eastern Australia. Mycopathologia.

[11] Joyce A., Gupta A.K., Koenig L., Wolcott R., Carviel J. (2019). Fungal diversity and onychomycosis: An analysis of 8,816 toenail samples using quantitative PCR and next-generation sequencing. J. Am. Podiatr. Med. Assoc..

[12] Hainsworth S., Lawrie A.C., Vanniasinkam T., Grando D. (2022). Metagenomics of toenail onychomycosis in three Victorian regions of Australia. J. Fungi.

[13] De Hoog G.S., Dukik K., Monod M., Packeu Stubbe D., Hendrickx Kupsch C., Stielow J.B., Freeke F., Gőker M., Rezaei-Matehkolaei A., Mirhendi H. (2017). Toward a novel multilocus phylogenetic taxonomy for the dermatophytes. Mycopathologia.

[14] Nweze E.I., Eke I.E. (2018). Dermatophytes and dermatophytosis in the eastern and southern parts of Africa. Med. Mycol..

[15] Sherman S., Goshen M., Treidgerman O., Ben Zion K., Carp M.-J., Maisler N., Binsky Ehrenreich I., Kimchi A., Lifshitz S., Smollan G. (2018). Evaluation of multiplex real-time PCR for identifying dermatophytes in clinical samples—A multicentre study. Mycoses.

[16] Nilsson K., Friberg M., Rollman O., Tano E. (2019). Impact of prolonged storage of clinical samples at 4 °C on the recovery of dermatophytes by culture or PCR analysis. J. Mycol. Med..

[17] Dvořák M.D., Hubálek Z., Otčenášek M. (1968). Survival of dermatophytes in human skin scales. Arch. Dermatol..

[18] Sinski J.T., Wallis B.M., Kelley L.M. (1979). Effect of storage temperature on viability of *Trichophyton mentagrophytes* in infected guinea pig skin scales. J. Clin. Microbiol..

[19] Kupsch C., Ohst T., Pankewitz F., Nenoff P., Uhrla S., Winter I., Gräser Y. (2016). The agony of choice in dermatophyte diagnostics–performance of different molecular tests and culture in the detection of *Trichophyton rubrum* and *Trichophyton interdigitale*. Clin. Microbiol. Infect..

[20] Pospischil I., Reinhardt C., Bontems O., Salamin K., Fratti M., Blanchard G., Chang Y.-T., Wagner H., Hermann P., Monod M. (2022). Identification of dermatophyte and non-dermatophyte agents in onychomycosis by PCR and DNA sequencing—A retrospective comparison of diagnostic tools. J. Fungi.

[21] Gupta A.K., Cooper E.A., Wang T., Lincoln S.A., Bakotic W.L. (2023). Single-point nail sampling to diagnose onychomycosis caused by non-dermatophyte molds: Utility of polymerase chain reaction (PCR) and histopathology. J. Fungi.

[22] Brillowska-Dąbrowska A., Nielsen S.S., Nielsen H.V., Arendrup M.C. (2010). Optimized 5-hour multiplex PCR test for the detection of tinea unguium: Performance in a routine PCR laboratory. Med. Mycol..

[23] Miyajima Y., Satoh K., Uchida T., Yamada T., Abe M., Watanabe S., Makimura M., Makimura K. (2013). Rapid real-time diagnostic PCR for *Trichophyton rubrum* and *Trichophyton mentagrophytes* in patients with tinea unguium and tinea pedis using specific fluorescent probes. J. Dermatol. Sci..

[24] Wang Q., Huang X., Yan Q., Chen R., Shao L., Li R., Song Y., Yuan X. (2024). Detection of pan-dermatophytes and *Trichophyton rubrum* using recombinase polymerase amplification-lateral flow dipstick assay. Mycopathologia.

[25] Heckler I., Sabalza M., Bojmehrani A., Venkataraman I., Thompson C. (2023). The need for fast and accurate detection of dermatomycosis. Med. Mycol..

[26] Mehlhorn C., Uhrlaß S., Klonowski E., Krueger C., Paasch U., Simon J.C., Nenoff P. (2024). Conventional and molecular diagnostics in onychomycosis-part 2: Molecular identification of causative dermatophytes by polymerase chain reaction and sequence analysis of the internal transcribed spacer region of ribosomal DNA. Dermatologie.

[27] Marin-Maldonado F., Pacheco-Torres A., Gustafson E. (2023). Comparative analysis of onychomycosis in Puerto Rico using molecular and conventional approaches. J. Med. Mycol..

[28] Uchida T., Makimura K., Ishihara K., Goto H., Taiiri Y., Okuma M., Fujisaki R., Uchida K., Abe S., Iijima M. (2019). Comparative study of direct polymerase chain reaction, microscopic examination and culture-based morphological methods for detection and identification of dermatophytes in nail and skin samples. J. Dermatol..

[29] Bergmans A.M.C., van der Ent M., Klaassen A., Bohm N., Andriesse G.I., Wintermans R.G.F. (2010). Evaluation of a single-tube real-time PCR for detection and identification of 11 dermatophyte species in clinical material. Clin. Microbiol. Infect..

[30] Ebihara M., Makimura K., Sato K., Abe S., Tsuboi R. (2009). Molecular detection of dermatophytes and nondermatophytes in onychomycosis by nested polymerase chain reaction based on 28S ribosomal RNA gene sequences. Br. J. Dermatol..

[31] Sánchez M.J.I., Pico A.M.P., Tejedor F.M., Sánchez M.J.I., Acevedo R.M. (2014). Using a polymerase chain reaction as complementary test to improve the detection of dermatophyte fungus in nails. J. Am. Podiatr. Med. Assoc..

[32] Taplin D., Zaias N., Rebell G., Blank H. (1969). Isolation and recognition of dermatophytes on a new medium (DTM). Arch. Dermatol..

[33] Navarro-Pérez D., Garcia-Oreja S., Tardáguila-Garcia A., León-Herce D., Álvaro-Afonso F.J., Lázaro-Martinez J.L. (2023). Microbiological culture combined with PCR for the diagnosis of onychomycosis: Descriptive analysis of 121 patients. Mycoses.

[34] Coloe S.C., Baird R. (2010). Dermatophyte infections in Melbourne: Trends from 1961/64 to 2008/09. Australas J. Dermatol..

[35] Shemer A., Daniel R., Kassem R., Geffen Y., Galili E. (2020). Cold sub-atmospheric and atmospheric pressure plasma for the treatment of *Trichophyton rubrum* onychomycosis: An in-vitro study. Dermatol. Ther..

[36] White T.J., Bruns T.D., Lee S.B., Taylor J.W. (1990). Amplification and sequencing of fungal ribosomal RNA genes for phylogenetics. PCR Protocols: A Guide to Methods and Applications.

[37] Stevenson A., Hamill P.G., Dijksterhuis J., Hallsworth J.E. (2017). Water-, pH- and temperature relations of germination for the extreme xerophiles *Xeromyces bisporus* (FRR 0025, *Aspergillus penicillioides* (JH06THJ) and *Eurotium halophilicum* (FRR2471). Microb. Biotechnol..

[38] Sneath P.H.A., Sokal R.R. (1973). Numerical Taxonomy.

[39] Felsenstein J. (1985). Confidence limits on phylogenies: An approach using the bootstrap. Evolution.

[40] Jukes T.H., Cantor C.R., Munro H.N. (1969). Evolution of protein molecules. Mammalian Protein Metabolism.

[41] Kumar S., Stecher G., Tamura K. (2016). MEGA7: Molecular Evolutionary Genetics Analysis version 7.0 for bigger datasets. Mol. Biol. Evol..

[42] Hainsworth S. (2022). Advances in Studies of Australian Dermatophytes and Tinea Unguium. Ph.D. Thesis.

[43] Debuysschere C., Blairon L., Cupaiolo R., Beukinga I., Tré-Hardy M. (2023). Clinical evaluation of a dermatophyte RT-PCR assay and its impact on the turn-around-time: A prospective study. Med. Mycol..

[44] Muir D., Pritchard R.C., Gregory J.D. (1984). Dermatophytes identified at the Australian National Reference Laboratory in Medical Mycology. Pathology.

[45] Ross I.L., Weldhagen G.F., Kidd S.E. (2020). Detection and identification of dermatophyte fungi in clinical samples using a commercial multiplex tandem PCR assay. Pathology.

[46] Heikkila H. (1996). Isolation of fungi from onychomycosis-suspected nails by two methods: Clipping and drilling. Mycoses.

[47] Hasegawa T. (1975). *Trichophyton rubrum* and *T*. mentagrophytes studied by freeze-etching. Sabouraudia.

[48] Farley D.L. (1921). The viability of ringworm fungi in dry cutaneous material. Arch. Dermatol. Syphilol..

[49] Bontems O., Hauser P.M., Monod M. (2009). Evaluation of a polymerase chain reaction-restriction fragment length polymorphism assay for dermatophyte and nondermatophyte identification in onychomycosis. Br. J. Dermatol..

[50] Nowicka D., Nawrot U., Wlodarczyk K., Pajaczkowska M., Patrzalek A., Pecak A., Mozdyniewicz P., Fleischer M. (2016). Detection of dermatophytes in human nail and skin dust produced during podiatric treatments in people without typical clinical signs of mycoses. Mycoses.

[51] Winter I., Uhrlaß S., Krüger C., Herrmann J., Bezold G., Winter A., Barth S., Simon J.C., Gräser Y., Nenoff P. (2013). Molekularbiologischer Direktnachweis von Dermatophyten im klinischen Material bei Verdacht auf Onychomykose und tinea pedis. Hautarzt..

[52] Zhao Y., Wang X., Lu C. (2024). Diagnostic values of ten methods in patients with onychomycosis: A network meta-analysis. Mycoses.

