# Supplementary material for: Specific Primers and Nested PCR Find Trichophyton rubrum Missed by Culture of Ground Toenails from Onychomycosis in Podiatric Patients in Eastern Australia
Source: J Fungi (Basel). 2025 Jul 14;11(7):520. doi: 10.3390/jof11070520 (PMC12295966; doi:10.3390/jof11070520)
Supplement: Supplementary file 1 [file jof-11-00520-s001.zip › jof-3702988-supplementary.pdf]

Supplementary data

Table S1. Cultures used during this research.

Name	RMIT Fungal Culture Collection (RFCC) number
Aspergillus thermomutatus	163
Epidermophyton floccosum	113(1), 113(2)
Microsporum canis	232, 236, 237
Paraphyton cookei	127
Trichophyton interdigitale	227, 374 (ATCC MYA-4439), 666 (SH70), 667 (SH171), 668 (SH180), 669 (SH227)
Trichophyton rubrum	164, 230, 371 (ATCC MYA-4438)
Trichophyton tonsurans	162

ATCC refers to culture numbers in the American Type Culture Collection. SH refers to isolate numbers assigned by Steven Hainsworth in Hainsworth et al. (2020).

Table S2. Closest matches of ITS sequences in Blast searches on NCBI using universal primers ITS5+ITS4.

GenBank Accession Number	Sequence searched	Closest match*	Cover (%)	% match
OP271463	232 Mca	LC317653 *M. canis*	99	99.45
OP271464	236 Mca	KC833579 *N. otae* (*M. canis*) YY01986877	99	99.31
OP271466	113 Ef	MT431956 *E. floccosum* 6000013/20	99	99.45
OP271468	162 Tto	LC317869 *T. tonsurans*	99	99.70
OP271471	163 Atherm	MK111645 *Aspergillus thermomutatus*	99	99.32
OP271467	227 Tmi	KC923427 *T. interdigitale* YY014374442	99	98.67
OP271470	230 Tr	KC923435 *T. rubrum* YY01436205	99	99.85
OP271469	164 Tr	KY427912 *T. rubrum* dermaiims14	99	99.41
OP271465	127 Pco	JN134139 *Paraphyton cookei*	99	99.41
N/A	R4 toenails	MZ337805 *T. rubrum* D4	95	99.37
N/A	R38 toenails	MZ337805 *T. rubrum* D4	97	98.78
N/A	R5 toenails	EF652036 *A. penicillioides* NRRL4548-T	99	98.69

*GenBank accession number, species name, strain name if given in record. T=Type strain. N/A = not applicable.

Table S3. Closest matches of sequences in Blast searches on NCBI using new specific primers.

**Primers**	**Sequence searched**	**Closest match***	**Cover (%)**	**ID (%)**
ITS5+TmiRev	227 Tmi-TmiRev	MK447609 *T. mentagrophytes* DSM108626	99	99.12
(not nested)	162 Tto-TmiRev	MN704387 *T. tonsurans* CEMM-01-3- 091	98	99.30
	113 Ef-TmiRev	MT431956 *E. floccosum* 6000013/20	98	99.52
Nested	227 Tmi-TmiRev	MK447609 *T. mentagrophytes* DSM108626	99	99.12
1TS5+ITS4->	R4- TmiRev	OM801501 *T. interdigitale* F10#	100	100
ITS5+TmiRev	R5-TmiRev	ON059700 *T. interdigitale* DERM RML12	100	100
	R33-TmiRev	MK447596 *T. interdigitale* DSM108421	99	99.47
	R8-TmiRev	OM801501 *T. interdigitale* F10	100	100#
	R9-TmiRev	MK447596 *T. interdigitale* DSM108421	99	99.47
	R35-TmiRev	MK447596 *T. interdigitale* DSM108421	99	99.47
	R34-TmiRev	MK447596 *T. interdigitale* DSM108421	99	99.65
Nested	230 Tr-Rev	MT623559 *T. rubrum* 211856/17	99	99.65
1TS5+ITS4->	R4-TrRev	MT623559 *T. rubrum* 211856/17#	99	99.97
ITS5+TrRev	R5-TrRev	MT623559 *T. rubrum* 211856/17	99	99.65
	R33-TrRev	MT623559 *T. rubrum* 211856/17	98	97.31
	R8-TrRev	KP326579 *T. rubrum* UZ1588_14	98	100.00
	R9-TrRev	MT623559 *T. rubrum* 211856/17	99	99.82
	R35-TrRev	MT623559 *T. rubrum* 211856/17	99	100
	R34-TrRev	MT623559 *T. rubrum* 211856/17	98	100
	R38-TrRev	MT623559 *T. rubrum* 211856/17	99	99.47
	R40-TrRev	MT623559 *T. rubrum* 211856/17	99	99.82

*GenBank accession number, species name, strain name if given in record. T=Type strain. N/A = not applicable.

Table S4. Relative discovery of dermatophytes in these ground toenails by different methods.

**Method used**	***Trichophyton interdigitale***	***Trichophyton rubrum***	***Epidermophyton floccosum***	***Paraphyton cookei***	**Reference**
Culture (no. of isolates)	53	1	0	1	Hainsworth et al. (2020)
Specific primers for:					
– *T. interdigitale*	+	–	±	–	This study
– *T. rubrum*	–	+	±	–	
Metagenomics of nails (mean % of sequence assemblies)	21.3	21.25	1.17	–	Hainsworth et al. (2022)
